# Exposure of Fluoride with Streptozotocin-Induced Diabetes Aggravates Testicular Damage and Spermatozoa Parameters in Mice

**DOI:** 10.1155/2019/5269380

**Published:** 2019-12-03

**Authors:** Manuel Sánchez-Gutiérrez, Evelia Martínez-Loredo, Eduardo Osiris Madrigal-Santillán, Gabriel Betanzos-Cabrera, Araceli Hernández-Zavala, María Angélica Mojica-Villegas, Jeannett Alejandra Izquierdo-Vega

**Affiliations:** ^1^Área Académica de Medicina, Instituto de Ciencias de la Salud, Universidad Autonoma del Estado de Hidalgo, Carretera Actopan-Tilcuautla, Ex-hacienda La Concepción S/N, San Agustín Tlaxiaca 42160, Hidalgo, Mexico; ^2^Tecnológico de Monterrey, Escuela de Ingeniería y Ciencias, Campus Querétaro, Epigmenio González 500, 76130 Querétaro, Mexico; ^3^Sección de Investigación y Posgrado, Escuela Superior de Medicina del Instituto Politécnico Nacional, Plan de San Luis y Díaz Mirón Col, Casco de Santo Tomas, Delegación Miguel Hidalgo, 11340 Ciudad de México, Mexico; ^4^Laboratorio de Toxicología de la Reproducción-Fertilidad, Escuela Nacional de Ciencias Biológicas, Instituto Politécnico Nacional, Av Wilfrido Massieu S/N, Colonia Adolfo López Mateos, Ciudad de México 07738, Mexico

## Abstract

Diabetes mellitus is the most common chronic disease worldwide that causes numerous complications, including male infertility. The prevalence of DM is 451 million people and estimated that would increase to 693 million in 2045. Fluorosis caused by drinking water contaminated with inorganic fluoride is a public health problem in many areas around the world. Previous studies have shown that fluoride exposure damages the male reproductive function. This study aimed to evaluate the fluoride sub-chronic exposure on the spermatozoa function in streptozotocin (STZ)-induced diabetic mice. After confirming diabetes by measuring blood glucose levels, the male mice received 45.2 ppm of fluoride added or deionized water. We evaluated several parameters in diabetic mice exposed to fluoride: standard quality analysis, the mitochondrial transmembrane potential (*ψ*m), the caspase activity in spermatozoa, urinary fluoride excretion, and histological evaluation in the testes. After 60 days of fluoride-exposure, diabetic mice, significantly decreased sperm quality (motility, viability, and concentration). Spermatozoa from fluoride-exposure in diabetic mice presented a significant decrease in *ψ*m and a significant increase in activity caspase 3/7. Urinary fluoride excretion was decreased in diabetic mice exposed to fluoride. Subchronic fluoride exposure of mice with STZ-induced diabetes aggravated testicular damage and the spermatozoa function.

## 1. Introduction

Fluoride is an abundant environmental pollutant widely existing in rocks, soil, water, food, and others [1]. Although the concentration of fluoride in water depends on every geographical location, the principal sources of fluoride in the human body are fluoride containing dental products and fluoridated water. Worldwide studies reveal that various regions of Africa, USA, Argentina, Bulgaria, China, Ethiopia, Iran, Korea, and Mexico have a high fluoride concentration leading to severe contamination of drinking water [2]. Recently, the U.S. PHS recommendation stated that the optimum concentration of fluoride in drinking water should be within a range of 0.7–1.2 ppm [3]. Fluorosis, a disease caused by depositions of fluoride in the body, affects the skeletal tissue and teeth [4], but also soft tissues, such as the liver [5], the kidney [6], the brain [7, 8] and the testes tissues [9]. Spermatozoa generated in the process of spermatogenesis are highly specialized cells, which functions in transporting and delivering the male genetic information to the descendant, the integrity of spermatozoa DNA, is a keystone of reproductive success which includes fertilization of the oocyte and embryonic development [10]. Several investigations suggest that fluoride exposure negatively impacts male reproduction to different levels, in the testes, causes several alterations in this organ affecting tissue structures, the presence of cellular apoptosis, the cell cycle and the structure of Leydig cells, altering the spermatogenesis process [9, 11–15]. Additionally, several studies have revealed that fluoride affects spermatozoa functions negatively; including morphology, motility, maturation, the acrosome reaction, capacitation, hyperactivation, and chemotaxis; thus, decreasing fertility [14, 16–22].

The world prevalence of Diabetes mellitus (DM) in adults reached 451 million people in 2017, and it estimated that would increase to 693 million people in 2045 [23]. DM is a well-known chronic metabolic disease characterized by prolonged hyperglycemia resulting from an alteration in the secretion or action of insulin. Uncontrolled diabetes leads to complications such as hyperglycemia with ketoacidosis or hyperosmolar nonketotic syndrome [24]. DM is the most common disease worldwide that affects patients' quality of life due to the several long-term complications of the disease that include retinopathy, nephropathy, and neuropathy autonomic, as well as detrimental effects on male reproductive function [24–26]. Male sexual dysfunction and impairments of male fertility at multiple levels by impairing the testicular endocrine and exocrine function or by disrupting the secretory function spermatogenesis, steroidogenesis, sperm maturation, impairment of penile erection and ejaculation are some of the leading secondary complications of DM [26–28]. This study aimed to evaluate the influence of fluoride subchronic-exposure on spermatozoa function in streptozotocin (STZ)-induced diabetes in mice.

## 2. Materials and Methods

### 2.1. Animals and Experimental Design

Male CD1 mice were obtained from the Institute of Health Sciences at the Autonomous University of Hidalgo (Hidalgo, Mexico). The animals were maintained according to the norms of the Institutional Ethics Animal Care and Use Committee (CIECUAL), under standard conditions with a 12 h/12 h light/dark cycle, constant temperature (22 ± 2°C) and humidity (50%). Food (Lab Diet® 5013) and water freely available in their home cages. The experimental procedures approved by CIECUAL of the Autonomous University of Hidalgo.

Twenty-four animals were randomly issued into four experimental groups as follows: group I (control), group II (STZ-induced diabetes), group III (fluoride-exposure), and group IV (STZ-induced diabetes plus fluoride exposure). Mice (45-day-old) were made diabetic by applying a single dose intraperitoneal injection of 150 mg/kg STZ dissolved in citrate buffer (pH 4.5). The tail vein blood glucose was measured after seven days of injection, and those animals with a blood glucose concentration ≥250 mg/dl were included in this study. At 60 days, the mean blood glucose in mice-STZ-treated was 460 mg/dl. Co-exposure with fluoride started once diabetes was confirmed in the animals. Control group mice received with deionized water, and the mice from groups III and IV received water-containing fluoride at 45.2 ppm during 60 days. The animals were kept for 60 days to assure more than one spermatogenic cycle in mice (approximately 40 days) [29].

### 2.2. Spermatozoa Quality

After 60 days of treatment, the mice were euthanized by cervical dislocation, the testis-epididymis-vas deferens complexes were dissected, and the spermatozoa were isolated by flushing the vas deferens and cauda epididymis lumens with one ml of phosphate-buffered saline (PBS, pH 7.4) at 37°C. Sperm parameters, including motility, viability, and concentration, were evaluated according to the method previously described under a light microscope at a magnification of ×400 [30]. Twenty microliters of sperm suspension were transferred on a clean glass slide. The sperm motility was assessed by counting the motile and immobile cells in five separate and random fields each slide by duplicate. Sperm count was evaluated by dilution of sperm suspension (1 : 20) in PBS. Ten microliters were transferred to the Neubauer chamber (HBG, Hamburg, Germany). Spermatozoa were manually counted, in duplicate, by light microscopy (Olympus Corp. Center Valley, PA USA), and data were expressed as million/ml. The spermatozoa viability was evaluated by mixing sperm suspension with an equal amount of (0.4%) trypan blue. The count of unstained and dead spermatozoa was performed under a light microscope (Olympus Corp. Center Valley, PA USA). For each mice sample,100 duplicate counted cells.

### 2.3. Spermatozoa Mitochondrial Membrane Potential

The mitochondrial membrane potential (*ψ*m) was measured with fluorophore JC-1 dye. JC-1 form monomers are emitting green fluorescence in mitochondria with low membrane potential. A 3 mM stock solution of JC-1 (Molecular Probes) in DMSO was prepared for mitochondrial membrane potential staining. Each sperm sample in PBS (5 × 10^6^/ml) was stained with 2.5 *μ*l of JC1 stock solution [31]. Fluorescence intensities were measured for 10,000 cells using a flow cytometer (FACSCalibur system, Becton Dickinson; Franklin Lakes, NJ).

### 2.4. Detection of Caspase 3/7 Enzymatic Activity

The detection of active Caspase 3 and 7 was carried out by Cell Event caspase 3/7 Green detection reagent, which is a four-amino acid peptide (DEVD), conjugated with a nucleic acid binding dye. After the activation of caspase-3/7 in apoptotic cells, the DEVD peptide is cleaved, enabling the dye to bind to DNA and produce a bright, fluorogenic response. For each spermatozoa sample, 1 ml of sperm suspension in PBS (10 × 10^6^ spermatozoa/ml) was incubated during 30 min at 37°C with caspase 3/7 reagent (2 *μ*M) and SYTOX (1 *μ*M) [32]. The fluorescence intensities were measured on 10,000 cells using a flow cytometer (FACSCalibur system Becton Dickinson; Franklin Lakes, NJ).

### 2.5. Fluoride Urinary Concentration

During the exposure period to fluoride, urine was collected three times at 0, 30, and 60 days of the study in a nonfasted state. For the urine collection, the animals remained in metabolic cages, and urine was collected over 24 h to quantify the fluoride concentration by a potentiometric method using an ion-selective electrode (Orion 9609). For its analysis, the urine was mixed with TISAB II (1 : 1). The electrode was calibrated at the standard range of 0.01–10 ppm [33]. All reading was duplicated.

### 2.6. Histology Analysis

The weight of testes was recordered to calculate the gonadosomatic index using the following formula: [gonada weight × body weight^−1^] × 100. The control testes samples and the diabetic fluoride-exposed mice were fixed with to 10% of formalin in phosphate-buffered saline (pH 7.4) and processed with conventional paraffin-embedding methods. Afterward, the paraffinized-tissue blocks were sectioned at 5 *μ*m and stained with hematoxylin and eosin (H&E). The count and diameter of seminiferous tubules were obtained from the tubular cross-sections that were more circular. Four cross-sections histological were evaluated per animal. The measurements were made with a high-definition camera mounted on a light microscope (Olympus Corp., Center Valley, PA, USA), at 10× lens and equipped with V.3.7. AmScope software (Myford Road, Irvine, CA, USA).

Moreover, Johnsen's criteria evaluated spermatogenesis was performed by analyzing twenty-five seminiferous tubules per animal. In this evaluation, a score is assigned to describe the spermatogenesis process quantitatively. Considering that the progressive degeneration of the seminiferous tubules presents the loss of mature spermatozoa and continues until the loss of spermatogonia, then to Sertoli cells. According to these criteria, a score of 9 or 10 indicates normal testicular histology, a score of 8 signifies hypo-spermatogenesis. A score of 3–7 represents maturation arrest, a score of 2 indicates Sertoli cells aplasia and, a score of 1 indicates tubular fibrosis. The samples were examined with phase-contrast microscopy at 40× magnification [34].

### 2.7. Data Analysis

The program Sigma Stat Statistics Analysis System version 4.0 was used to analyze the data statistics. The one-way ANOVA followed by a Bonferroni correction to evaluate pairwise differences. A *p*-value <0.05 was considered significant.

## 3. Results

### 3.1. Fluoride Exposure under Conditions of STZ-Induced Diabetes Aggravated Deterioration in Sperm Quality

In general, at 60 days, the mean body weight in the fluoride plus STZ group decreased significantly 1.40-fold and 1.16-fold, compared with the control and fluoride-exposed groups respectively. Also, there was no significant difference in body weight compared with the STZ group (Table 1). Next, we analyzed the overall quality of spermatozoa according to several parameters summarized in Table 1. The spermatozoa motility significantly decreased in the fluoride plus STZ group (3.8-fold) compared with the control group. Likewise, their motility decreased (2.39-fold and 2.47-fold), compared with the STZ group and exposed to fluoride, respectively. The sperm concentration also significantly reduced in the fluoride plus STZ (2.6-fold) in comparison to the control group, and a significant decrease of the sperm concentration of (2.55-fold and 2.9-fold), compared with the STZ group and exposed to fluoride, respectively. As shown, the sperm viability decreased significantly in the fluoride plus STZ group (2.6-fold) compared with the control group and (1.77-fold and 1.46-fold), compared with the STZ group and exposed to fluoride respectively. The gonadosomatic index decreased significantly in the fluoride plus STZ group (1.57-fold), compared with the control group and (1.26-fold), compared to the fluoride-exposed group. Additionally, we performed the histological evaluation with the Johnsen's testicular score. As shown in Table 1, the control testes demonstrated the typical testicular architecture and regular seminiferous tubular morphology including the outermost layer of Sertoli cells and spermatogonia, the middle layer of spermatocytes and the innermost layer of spermatozoa, which are indicative of normal spermatogenesis. However, in the testes of diabetic mice exposed to fluoride, we observed a decrease in size and number of the seminiferous tubules, irregular tubules and a lower number of spermatogonia and spermatocytes that result in a significant reduction in the Johnsen's testicular score, revealing the extent of testicular impairment in these conditions.

### 3.2. Fluoride Exposure under Conditions of STZ-Induced Diabetes Caused a Higher Decrease in Δ*Ψ*m Accompanied by an Increase in the Activation of Caspases

**Figure 1:**
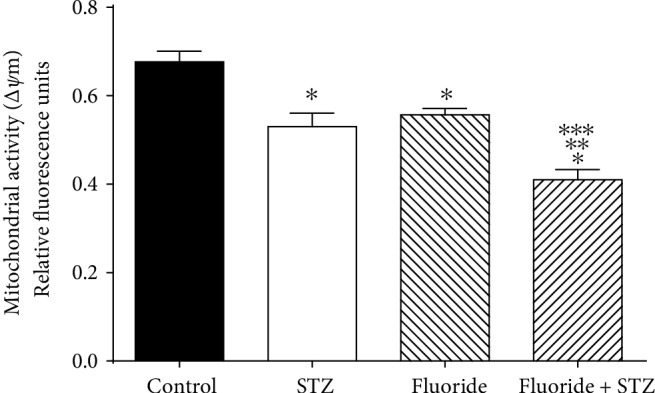
Effect of subchronic fluoride exposure in STZ-induced on Δ*Ψ*m. Δ*Ψ*m in spermatozoa was determined using invitrogen MitoProbe JC-1 assay kit, and the fluorescence was measured by flow cytometer. Results represent three independent experiments performed by triplicate. Values are means ± SD of 6 mice per group. (^∗^*p* < 0.05 vs. control group; ^∗∗^*p* < 0.05 vs. STZ group; ^∗∗∗^*p* < 0.05 vs. fluoride group).

**Figure 2:**
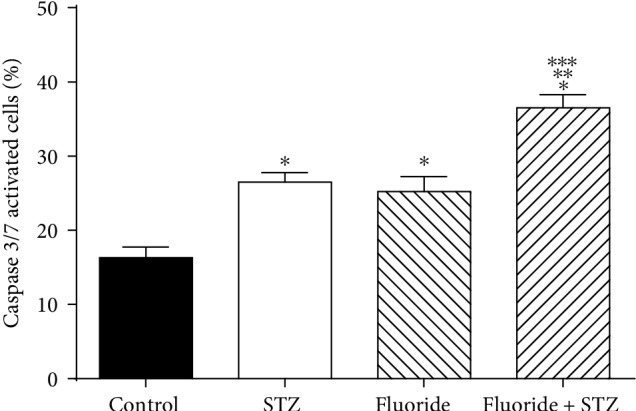
Effect of subchronic fluoride exposure in STZ-induced on caspase 3/7 activity. Detection of spermatozoa with activated caspases by cytometry flow using invitrogen cell event caspase 3/7. Subchronic fluoride exposure in STZ-induced diabetes leads to a greater increase in caspase 3/7 activated. Values are means ± SD of 6 mice per group (^∗^*p* < 0.05 vs. control group; ^∗∗^*p* < 0.05 vs. STZ group;^∗∗∗^*p* < 0.05 vs. fluoride group) (ANOVA and Bonferroni).

The mitochondrial membrane potential (Δ*Ψ*m) evaluation is an indicator of sperm quality. We evaluated the mitochondrial activity using fluorophore JC-1. The results have shown that the STZ plus fluoride group significantly reduced the Δ*Ψ*m of spermatozoa (1.65-fold) compared with the control. Also, the mitochondrial activity decreased (1.29-fold) compared with the STZ group, and in the same way, (1.34-fold) compared with the fluoride group (Figure 1). We evaluated the apoptosis using a Cell Event caspase-3/7 Green Detection Reagent. The results showed that STZ plus Fluoride group significantly increased the activation of caspases 3/7 (2.23-fold) compared with the control, and increased (1.62-fold) compared with the STZ group, and in the same way, (1.54-fold) compared with the fluoride group (Figure 2).

### 3.3. Fluoride Exposure under Conditions of STZ-Induced Diabetes Caused a Decrease in Urinary Fluoride Excretion

**Figure 3:**
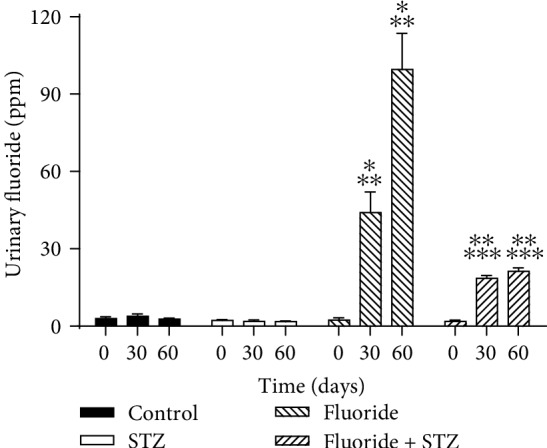
Urinary fluoride concentration during treatment for all groups. Control group, STZ group, fluoride group, fluoride in STZ-induced diabetes. Values are means ± SD of 6 mice per group. (^∗^*p* < 0.0001 vs. control group; ^∗∗^*p* < 0.0001 vs. STZ group;^∗∗∗^*p* < 0.0001 vs. fluoride group) (ANOVA and Bonferroni).

Figure 3 shows the urinary fluoride concentration in mice exposed to fluoride + STZ and control groups during 60 days. Suddenly, a significant reduction (4.68-fold and 2.37-fold) in the urinary fluoride concentration at 30 and 60 days caused the fluoride exposure to STZ-induced diabetic mice in comparison with the fluoride group.

## 4. Discussion

This study aimed to assess the effect of a fluoride subchronic-exposure on impairments caused in spermatozoa due to the exposure of fluoride in STZ-induced diabetes mice. Several studies have shown that fluoride exposure has a negative effect on male reproduction, altering sperm quality, epidydimal maturation, capacitation, acrosome reaction, damage to DNA integrity, and fertilization [9, 16–21, 35]. Interestingly, the consumption of fluoride through drinking water in the population is associated with the increase in the incidence and prevalence of diabetes [36]. Besides, repeated exposure to fluoride has been shown to increase serum glucose in animals with STZ-induced diabetes [37]; which could be caused by reduced secretion of insulin in pancreatic beta cells [38].

On the other hand, both induced diabetes and diabetes disease have demonstrated adverse effects on sperm quality and sperm DNA integrity [26, 27, 39–41] . Due to the above, it is interesting to study the reproductive effects of fluoride exposure in diabetic conditions. An earlier study has documented that the exposure of STZ-induced diabetic animals to fluoride at high concentrations (270 ppm of F), aggravates the damage caused in testes [42]. In the present study, we used 100 mg/L of fluoride in drinking water, corresponding to 45.2 mg fluoride/L for 60 days) based on the LD50 value of 54.4 mg fluoride ion/kg body weight in male mice [43].

In the same study, a subchronic fluoride exposure under diabetic conditions negatively aggravated the spermatozoa quality (motility, viability, and concentration) compared to the diabetic and fluoride groups. Sperm motility is dependent upon the availability of energy obtained through ATP hydrolysis produced by oxidative phosphorylation [44]. Previous studies have shown that the exposure to fluoride significantly reduces the level of ATP in spermatozoa [16, 17], due to alterations in the complex III and IV of the electron transport chain, accompanied by pathological changes in the ultra-structure of mitochondria [45, 46], besides the alteration in spermatozoa mitochondrial DNA copy number [44]. A mammalian sperm mitochondrial plays a crucial role in ATP production, calcium homeostasis osmotic regulation, production of reactive oxygen species (ROS), apoptosis, and others [47]. We also evaluated the sperm Δ*Ψ*m, which wide use characterizes the functional status of mitochondria and also is considered as a potential regulator and indicator of spermatozoa motility and hence is related to male fertility [31, 48]. Our results showed a higher decrease in Δ*Ψ*m in spermatozoa exposed to fluoride under diabetic conditions. Several studies have shown that fluoride exposure increases the generation of superoxide anion and other ROS [16, 49–51], and it is well-known that it significantly decreases the activity of antioxidant enzymes like superoxide dismutase (SOD) and catalase (CAT) in testes and spermatozoa [16, 52] and also decreases the activity and mRNA of SOD and CAT in epididymis [53]. Oxidative stress is an accepted mechanism of fluorosis in male infertility.

Apoptosis is a regulated cell death program that is a trigger via the extrinsic pathway, which implicates the activation of the cell surface death receptors, or the intrinsic pathway, which involves mitochondrial outer membrane permeabilization, or the apoptotic signaling caused by endoplasmic reticulum stress [54, 55]. Apoptosis plays an essential role in the spermatogenesis and sperm maturation [56]; however, abnormal apoptosis resulting from the mitochondrial pathways, alterations in testes can also negatively affect spermatogenesis and sperm count [57]. Apoptosis markers can be used to assess the capability of fertilization in spermatozoa [58]. In this study concerning apoptosis, a greater significant increase in the activation of caspase 3/7 in spermatozoa was observed in the subchronic fluoride exposure under diabetic conditions. Previously, it had been evidenced a significant increase in protein expressions of cytochrome c and active caspase-3 in spermatozoa of mice exposed to fluoride explaining the high ratio of apoptosis. Evidence shows that fluoride exposure before pre-pregnancy, during gestation, birth, and post-puberty, results in testicular endoplasmic reticulum stress and inflammatory response, as well as oxidative stress and germ cell apoptosis mediated by mitochondrial pathways and upregulation of FAS expression in testes [52, 59]. In this study, we observed a significant alteration in testicular histology in the subchronic fluoride under diabetic conditions. Previous studies showed that fluoride exposure causes damage to function and testicular structure related to oxidative stress and apoptosis [19, 59, 60].

Likewise, several alterations caused by the exposure to fluoride in male reproduction have been corroborated by microarray analysis and real-time RT-PCR, 63 down-regulated genes, which are involved in several sperm biological processes including signal transduction, oxidative stress, apoptosis, electronic transport chain, glycolysis, chemotaxis, spermatogenesis, and spermatozoa capacitation [45]. Recently, the alteration of several miRNAs involved in protease inhibitor activity, apoptosis, ubiquitin-mediated proteolysis, and signaling pathways of calcium, JAK-STAT, MAPK, p53, Wnt, have been evidenced; which were proved to be directly related to the sperm quality in reproductive toxicity caused by fluoride exposure [61].

Additionally, fluoride causes degeneration and necrosis of the tubular cells, renal tubular hyaline casts, and glomeruli swelling, accompanied by alterations of renal function caused by renal oxidative damage [62]. Also, alterations in renal functions have evidenced in children with dental fluorosis [63]. In the present study, fluoride exposure in diabetes conditions in mice decreased the excretion of urinary fluoride after 30 days of exposure. Since fluoride is eliminated through kidneys, the renal function insufficiency would impair clearance of fluoride suggesting that these conditions, the adverse effects of chronic fluoride exposure may be aggravated. Earlier studies have documented that the exposure to fluoride of STZ-induced diabetes animals aggravates the damage caused in the kidney [37].

On the other hand, DM is a disease that affects the duration and quality of life due to various intrinsic complications. DM can affect spermatogenesis process in multiple ways [28], that involve the impaired in hypothalamic-pituitary-gonadal axis function, a reduction in testosterone levels [64–66] and adverse outcomes in mating rate, fertility and number of litters [66, 67]. Oxidative stress play a vital role in the pathogenesis of male reproduction caused by DM through hyperglycemia that an increase of ROS production, decrease antioxidant defense, and initiate apoptosis, among other deleterious effects [68]. Interestingly the level of testosterone protects the human endothelial cells from advanced glycation end products (AGEs)-induced apoptosis mediated by caspase-3 and Bax/Bcl-2 [69]. Previously, it has shown that DM cause an alteration in quality spermatozoa (motility, viability, count, morphology), and an increase in DNA fragmentation, and impaired mitochondrial function [28, 39, 40, 70, 71]. Recently, the proteomics analysis in diabetic human semen showed an increase of several proteins associated with mitochondrial metabolic alterations, at the level of ATP synthase, cytochrome c oxidase, and NADH dehydrogenase that confirms the impaired mitochondrial function [72]. Interestingly, STZ-induced diabetes male mice altered the spermatozoa quality and expression patterns in offspring testes of two subsequent generations [71].

Recently it has been shown that co-exposures with fluoride with sulfur dioxide worsen on brain and kidney toxicity [73, 74]. In this study, similar alterations in spermatozoa quality, mitochondrial impaired function, apoptosis increasing have previously evidenced in the fluoride and diabetes exposure at reproductive level; however, their combination aggravates the spermatozoa function.

## 5. Conclusion

Results of this study suggest that the exposure of mice in diabetic conditions to fluoride aggravates the spermatozoa function, decreasing the mitochondrial membrane potential, increasing apoptosis and worsening histological changes of testicular cells. Besides, the diminution found in male quality parameters could be a reflection of the impairment in spermatogenesis by the co-exposure to fluoride and DM. Further studies is required, to characterize the mechanism by which the combined effect of fluoride in diabetic conditions exert a great toxic effect.

## Figures and Tables

**Table 1:** Assessment of spermatozoa parameters.

Sperm parameters	Control	STZ	Fluoride	Fluoride + STZ
Motility (%)	88.66 ± 3.82	55.83 ± 13.57^a^	57.83 ± 7.75^a^	23.33 ± 22.50^a,b,d^
Sperm concentration (10^6^/ml)	62.12 ± 11.43	40.43 ± 10.82^a^	46 ± 8.02^a^	15.81 ± 13.24^a,b,d^
Viability (%)	86 ± 1.09	57.33 ± 8.93^a^	48.5 ± 6.74^a^	33 ± 23.84^ab^
GSI (%)	0.592 ± 0.041	0.406 ± 0.018^a^	0.476 ± 0.065^a^	0.375 ± 0.072^a,d^
Body weight (g)	49.17 ± 1.74	35.06 ± 3.82^a^	30.56 ± 3.90^a^	42.25 ± 1.92^a,d^
Number of seminiferous tubules	290 ± 13.3	259 ± 8.2^a^	268 ± 7.2^a^	251 ± 9.1^a,b^
Seminiferous tubule diameter (mm)	0.23 ± 0.020	0.19 ± 0.017^a^	0.20 ± 0.019^a^	0.15 ± 0.015^a,b,d^
Histology (Johnsen's score)	9.14 ± 1.81	7.33 ± 2.27^a^	8.01 ± 1.61^a,d^	6.41 ± 2.17^a,b^

Values are means ± SD. ^a^(*p* < 0.05 vs. control); ^b^(*p* < 0.05 STZ vs. fluoride + STZ); ^c^(*p* < 0.05 STZ vs. fluoride); ^d^(*p* < 0.05 fluoride + STZ vs. fluoride) (ANOVA and Bonferroni).

## Data Availability

The data used to support the findings of this study are available from the corresponding author upon request.
